# Contributions to the Natural History of the United States

**Published:** 1855-09

**Authors:** 


### CONTRIBUTIONS TO THE NATURAL HISTORY OF THE UNITED STATES.

Prof. Agassiz is publishing by subscription the results of his researches in the Natural History of the United States. These inquiries are profound and original, and the results cannot be otherwise than interesting to all liberal cultivators of medical science. We insert below so much of Prof. A.'s Circular as is necessary to explain the aim and scope of his proposed work, which, we are warranted in saying, will reflect honor upon the science of our country:

"From a careful estimate of the materials I have now on hand, I am satisfied I shall be able to include the most valuable part of my investigations in ten quarto volumes; each volume containing about three hundred pages, with at least twenty plates. I therefore now open a subscription for such a work, in ten volumes, quarto, in cloth binding, at the price of twelve dollars each vol., payable on delivery. Each volume shall be complete in itself, containing one or several independent monographs; so that, if any unforseen difficulties should interrupt the publication of the whole, the parts already published shall not remain imperfect. As far as possible, I shall always select first such of my papers as contain the largest amount of new matter, or may contribute most directly to the advancement of science. Having devoted the greatest part of my time to the investigation of the embryonic growth of our animals, I shall make a beginning with the embryology of our turtles, several of which I have traced through all their changes. I trust this monograph will afford our medical students a fair opportunity of making themselves familiar with the modern results of one branch of physiology, which has the most direct bearing upon their science, and for which the different species of the family of turtles, found in every part of the United States, will afford them a bettor opportunity even than the artificial breeding of hen's eggs. Moreover, the extent of my embryological researches, covering, as they do, all the classes of the animal kingdom, will furnish, I trust, a new foundation for a better appreciation of the true affinities, and a more natural classification of animals. I forsee the possibility, upon this basis, of determining, with considerable precision, the relative rank of all the orders of every class of animals, and of furnishing a more reliable standard of comparison between the extinct types of past geological ages and the animals now living upon earth.

"It is a matter of course, that a work like this, illustrated by a large number of plates cannot be published without a liberal and extensive patronage. As it has been prepared solely with the view of throwing additional light upon the wonderful diversity of the animal creation of this continent, its structure, and its general relation to that of the other parts of the world, without the slightest hope of compensation for myself, I trust I may meet with the approbation of those conversant with the importance of the subject, and receive sufficient encouragement from the enlighted part of the community to enable me to bring to a successful close an undertaking upon which I enter now, and in this form, for no other purpose than to contribute my share towards increasing the love of nature among us.

"As the printing of this work cannot begin till a sufficient guarntee is secured for the publication of the whole, I take the liberty of making an appeal to the lovers of science to send to the publishers their own subscriptions, and such others as they may procure, as soon as convenient, and, if possible, before the first of August next, that I may be able to proceed at once with a work which, relating to animals peculiar to America, I wish to make, in every respect, an American contribution to science, fostered and supported by the patronage of the community at large.

"To render this work more generally accessible, it is intended to publish at the rate of about one volume a year. Such an arrangement will bring the whole within the reach of every student of Natural History, and of every friend of the progress of science in the country. The periods of publication, however, cannot be more definitely fixed, because the required uniformity of execution of the plates, to which particular attention will be paid, will demand that they all be intrusted to the same artist who has drawn on stone most of the plates of my former works.

"*Cambridge*, May, 1855."

